# Case of Cirrhosis of Liver and Kidneys

**Published:** 1878-04

**Authors:** C. F. Smolt


### CASE OF CIRRHOSIS OF LIVER AND KIDNEYS.

By C. F. Smolt, M. D.

On Nov. 27, 1877, I was called to visit Hattie S., aged 37. Her health had never been good, though she had not suffered from any definite disease. Her recovery had been slow after each of her confinements (the last of which had occurred May 20, 1877). On June 4, one of her children was attacked with scarlet fever, and subsequently three others developed it. During their illness the patient was their only nurse, and she was then herself confined to bed for four weeks, suffering from a disease whose name she did not know. She remembers simply that she had several chills and a continuous fever. After that time her breathing became more rapid; her skin acquired an ashen hue; she became subject to a short, hacking cough; her strength gradually failed. Nov. 22 she first noticed slight swelling of the feet.

I found the patient weak and emaciated; the pulse was rapid and feeble; there was a mitral regurgitant murmur; dyspnœa, apparently due largely to the distension of the abdomen with gas. The urine contained about 30 per cent. of albumen and numerous epithelial casts. The dropsy gradually extended up to the body. Slight arterial hemorrhages from the lungs occurred Jan. 17th and 21st. Subsequent to the latter date the patient was unable to leave her bed, though compelled to sit upright. Several "sinking spells" occurred during the week preceding death, which occurred Feb. 6.

Post mortem examination revealed a considerable quantity of serous liquid in the left pleural sac; the lower lobe of the right lung was consolidated; the abdomen contained but little liquid. The kidneys were small and dense; the cortical portion diminished in thickness.

*Microscopy.* — Upon microscopic examination, the tubules were observed to be of variable size, some greatly dilated and stuffed with epithelium, others contracted and almost empty. Perhaps the most striking feature was the thickness of the connective tissue constituting the capsules of the malpighian bodies and the outer coat of the vessels. The tubular epithelium was highly granular from the presence of fat, which had in many instances gathered into drops, which were visible both within and outside of the cells.

The malpighian tufts were generally much decreased in size, and were surrounded, as were the vessels, by a small-celled infiltrate. W. T. B.

---

